# Supplementary material for: Familial Aggregation of Endemic Congenital Hypothyroidism Syndrome in Congo (DR): Historical Data
Source: Nutrients. 2020 Oct 2;12(10):3021. doi: 10.3390/nu12103021 (PMC7601371; doi:10.3390/nu12103021)
Supplement: Supplementary file 1 [file nutrients-12-03021-s001.zip › Article S1 Vanderpas J 1994 Chpt 21 in The Damaged Brain of Iodine Deficiency.pdf]

A Franklin Institute Symposium Publication

from

"THE DAMAGED BRAIN OF IODINE DEFICIENCY: Cognitive, Behavioral, Neuromotor, and Educative Aspects"

May 4–5, 1993

The Franklin Institute
Benjamin Franklin Parkway at 20th Street
Philadelphia, Pennsylvania, U.S.A. 19103-1194

Copyright © COGNIZANT COMMUNICATION CORPORATION 1994

No part of this publication may be reproduced, stored in a retrieval system or transmitted in any form or by any means: electronic, magnetic tape, mechanical, photocopying, recording or otherwise without permission in writing from the publisher.

The Publisher and the Publisher's agents represent that the data provided were formulated with a reasonable standard of care. Except for this representation, the Publisher makes no representation or warranties, expressed or implied.

COGNIZANT COMMUNICATION OFFICES:

U.S.A. 3 HARTSDALE ROAD, ELMSFORD, NEW YORK, 10523-3701

AUSTRALIA P.O. BOX 352 CAMMERAY, NWS, 2062

JAPAN c/o OBK T's BLDG 3F, 1-38-11 MATSUBARA, SETAGAYA-KU TOKYO

Library of Congress Cataloging-in-Publication Data

The damaged brain of iodine deficiency: neuromotor, cognitive, behavioral , and educative aspects / edited by John B. Stanbury
p. cm.
Includes bibliographical references and index.
ISBN 1-882345-03-7
1. Iodine deficiency diseases--Congresses. 2. Cretinism--Nutritional aspects--Congresses. 3. Brain--Diseases--Nutritional aspects--Congresses. 4. Cognition disorders--Nutritional aspects--Congresses. I. Stanbury, John B. (John Burton), 1915-
[DNLM: 1. Brain--physiopathology. 2. Iodine--deficiency. 3. Cognition--physiology. 4. Psychomotor Performance. WL 300 D154 1994]
RA645.I55D36 1994
616.8 ' 0471--dc20
DNLM/DLC
for Library of Congress 94-22998
CIP

Printed in the United States of America

Printing: 1 2 3 4 5 6 7 8 9 10 Year: 1 2 3 4 5 6 7 8 9 10

Endemic Neonatal, Infantile, and Juvenile Hypothyroidism in Ubangi, Northern Zaire: Clinical Consequences and Prevention

J. Vanderpas and C.H. Thilly

Laboratoire d'Analyses Médicales, Hôpital Ambroise Paré
7000 Mons, Belgium

Biochemical hypothyroidism (TSH > 50 mU/L) affects one fourth of the neonates in the Ubangi area of northern Zaire. Goiter prevalence in their mothers is >75%, and the prevalence of endemic cretinism varies from 1 to 10% in various villages (1).

Biochemical infantile and juvenile hypothyroidism affects one fourth of the infants (<12 months) and one third of the children (>2 years) in the same area (2).

The question arises, then, concerning the efficiency of iodized oil administration to the pregnant mother on the prevention of neonatal, infantile, and juvenile hypothyroidism in this severe goiter endemia.

From 1973 to 1980, a placebo-controlled, longitudinal, randomized study was conducted in this area to assess the safety and the efficiency of iodized oil administration to the mother during pregnancy. Up to now, only the data on the prevention of neonatal hypothyroidism have been published (1). In this first part, we analyze the biochemical follow-up data of the thyroid function in the children of this study. We focus the analysis on the effects of iodine supplementation to the pregnant mother (during the last two trimesters of pregnancy) on the biochemical parameters of her newborn, infant, and child.

METHODS

Pregnant women (n = 983) were recruited at their first prenatal clinics at Karawa hospital, Ubangi from February 1973 to March 1980 (Table 1). They originated from the city of Karawa (n = 187) or from the rural villages surrounding Karawa (n = 796). Iodized oil (1 cc Lipiodol IM, treated group) or placebo (control group) was administered to every woman in a randomized allocation (closed envelopes containing the nature of treatment). At entry in the study, identification, clinical items (goiter volume in the mother according to WHO classification, uterine height) were recorded by a registered nurse (R.N.) and biological samples of blood and urine were collected from the mother to determine the serum T_4, T_3, and TSH concentrations and the urine iodine concentrations. For the women recruited in the study and delivering at Karawa hospital, the thyroid status of the mother (clinical examination) and some clinical parameters in the neonates (height, weight, obvious clinical problems) were recorded; at delivery, a blood sample of the mother and of the neonate (cord blood) and a urine sample of the mother were obtained to perform biological analysis. Follow-up data on the evolution of the children (mortality, goiter, cretinism, weight, height) were obtained from August 1973 to March 1980 during 11 visits at Karawa and in the villages around Karawa by six examiners (M.D.).

Biochemical thyroid status was determined during four visits by serum analysis in two cases (April 1974 and March 1980) and by collection

Table 1. Dates of Survey, Number of Women Included in the Longitudinal Karawa Study on Iodized Oil Supplementation During Pregnancy (Control Group/Treated Group), Number of Biological Samples Collected at these Different Times, and Number of Psychomotor Tests in the Children of the Study

Date of Survey	Recruitment and Follow-Up at Delivery Feb. 1973–Mar. 1980	Follow-Up Surveys		
		Mar.–Apr. 1974	Jan.–Feb. 1979	Jan.–Mar. 1980
Mothers recruited	484/499	118/133	409/412	484/499
Mother's serum				
Prenatal	389/411			
Delivery	235/246			
Child's serum				
Cord Serum	195/199			
<1 year-old		31/35	10/7[a]	57/66
1–4 year-old			54/58[a]	54/61
5–7 year-old			34/58[a]	68/89
Mother's urine				
Prenatal	381/389			
Delivery	232/232			
Child's Urine				
<1 year-old			9/4[a]	40/60
1–4 year-old			43/53[a]	40/40
5–7 year-old			28/48[a]	63/82
Psychomotor tests				175/218

[a]Blood sample collected on filter paper.

of blood on filter paper for analysis in the two other cases (January 1979 and July 1979). To prevent thyroid hormone measurements at too short intervals in the same child, the hormonal data of July 1979 are discarded for this presentation. Urinary samples were obtained during three visits (January 1979, July 1979, and March 1980). The examiner was unaware of the treatment allocation. Follow-up data were obtained at least once in 345 children of the treated group and in 315 children of the control group.

The description of the biochemical methods used was published previously (3). Concerning the filter papers, only total T_4 and TSH were determined on these samples.

RESULTS

Figure 1 (top) compares the distribution of urinary iodine concentrations in urinary samples just before treatment in pregnant women included in the study (control group: $n = 381$; treated group: $n = 389$). The two groups of mothers are similar according to the urinary iodine excretion, with 65% (500/770) women under the threshold defining a deficiency state (<5 μg/dL) and 25% (193/770) women in a severely deficient state (<2 μg/dL). At delivery (Figure 1, bottom), the distribution of iodine concentration in the control group is not distinguishable from that observed before treatment with 69% (160/232) of the women excreting less than 5 μg/dL iodine in the urine; however, in the treated group, iodine supplementation involves a decrease of this proportion to 8% (19/232).

Figure 2 describes the evolution of urinary iodine concentration in infants and children of the treated and the control group. In the control group, the mean urinary iodine concentration was systematically below 5 μg/dL during each age period, with small, nonsignificant changes of the mean levels between 1.5 and 3.6 μg/dL. In the treated group, mean urinary iodine concentration was in a normal elevated range during the first year (15.5 μg/dL); it decreased under the threshold of 5 μg/dL during the second year; and the evolution was similar to that of the control group after 3 years.

Figure 3 describes the evolution of the prevalence of abnormal hormonal values in the control group and in the treated group of children during three age periods. In the control group, the prevalence of elevated TSH (>10 mU/L) remained stable and elevated (0–24 months: 46%, 55/120; 24–36 months: 49%, 19/39) during the first 3 years. It increased significantly to 59% (88/150) after 4 years ($p < 0.05$ vs. children less than 36 months. In the treated group, during the first 2 years, the prevalence of elevated TSH values (5%, 7/138) was markedly lower than in

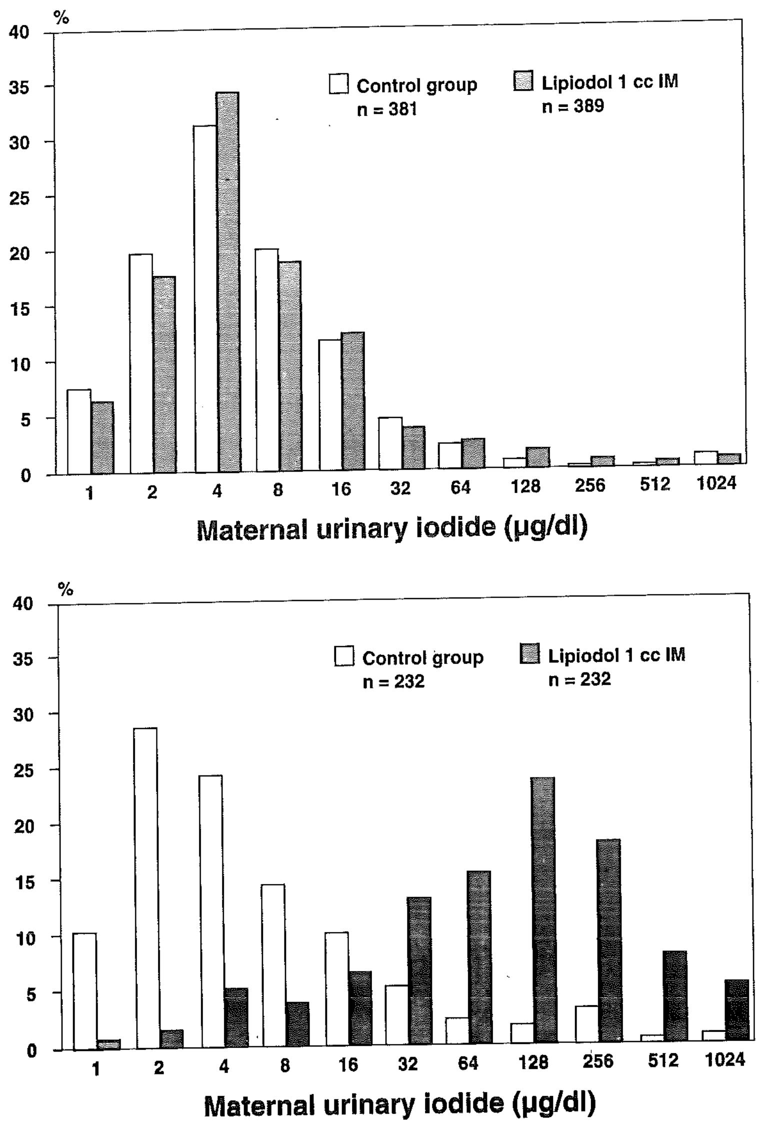

Figure 1. Top: distribution of the urinary iodide concentrations before treatment (at entry in the study) in the control mothers and in the treated mothers. Bottom: distribution of the urinary iodide concentrations in the same mothers at delivery, in the control mothers, and in the treated mothers.

the control group for the same age period ($p < 0.001$). During the third year, this prevalence increased to a level (41%, 19/46) similar to that observed in the control group, and it increased significantly after 3 years (71%, 137/193; $p < 0.001$ vs. treated group less than 36 months) at a level similar to that attained in the control group.

Although elevated serum TSH associated with low serum T_4 was frequent in the control group (between 23 and 57% depending on the age group) and in the treated group after 2 years (between 28 and 65% depending on the age group), the serum T_3 was abnormally low in a minority of cases (9%, 17/198, in the control group, 0–84 months and 8%, 9/114 in the treated group after 36 months).

Table 2. Percentage of Elevated Serum TSH During Childhood

Cord Serum TSH	Child's Serum TSH > 10mU/L	
<20 mU/L	26% (11/43)	
≥20 mU/L	67% (24/36)	RR = 2.6 (1.5–4.6)
		$\chi^2 = 13$ ($p < 0.001$)

Data are for 1–7 year-old children according to normal or elevated cord serum TSH in the same children. Karawa study, neonates, and children of the control group, RR, relative risk; χ^2, chi-square test.

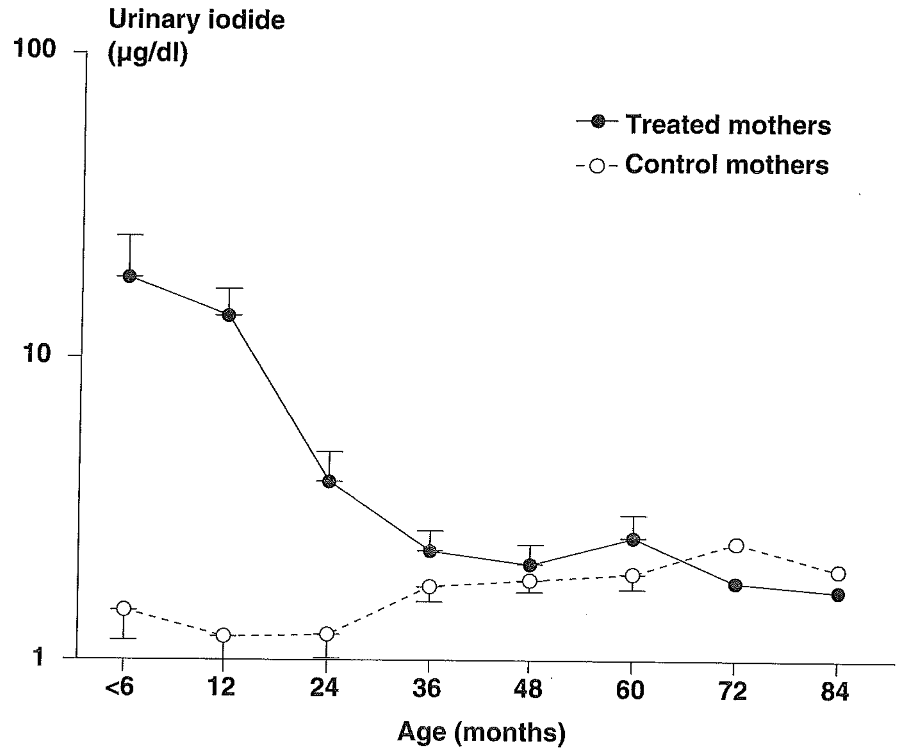

Figure 2. Evolution of the urinary iodide concentration in children of treated mothers (geom. mean + 1 SEM) and in children of control mothers (geom. mean - 1 SEM).

Table 2 compares the frequencies of abnormal serum hormonal concentrations in children of the control group (TSH > 10 mU/L) according to the serum hormonal concentrations at birth. Neonates with a cord serum TSH ≥ 20 mU/L have a 2.6-fold greater risk of having an elevated serum TSH during childhood than neonates with a cord serum TSH < 20 mU/L ($p < 0.001$). It is also notable that 12 of the 36 neonates with elevated TSH recovered a normal serum TSH during childhood (in the absence of iodine supplementation). Conversely, 11 of the 43 neonates in the control group with a normal cord serum TSH at birth (TSH < 20 mU/L) developed infantile or juvenile hypothyroidism at least at the time of the survey during their first 7 years.

The prevalence of clinically defined cretinism was relatively low as compared to the prevalence of biochemical hypothyroidism. In the treated group of the longitudinal Karawa study on iodized oil supplementation during pregnancy, 345 children were seen at least once during the 11 surveys in the villages, and nine of them were clinically classified as cretins (2.6%), with biochemical confirmation of hypothyroidism. A clinical examination was available under the age of 2 years in six cases; only one of them was diagnosed as cretin before 2 years. In the control group of the same study, 314 children were seen at least once during the 11 surveys in the villages, and 26 of them were clinically defined as cretins (8.3%), with biochemical confirmation of hypothyroidism. A clinical examination was available under the age of 2 years in 23 cases; 17 of these 23 cases were already diagnosed as hypothyroid cretins at that age (a manuscript with a more complete description of these cases of cretinism is in preparation).

DISCUSSION

The administration of iodized oil (1 cc Lipiodol IM) to the pregnant woman during the last two trimesters prevents neonatal and infantile hypothyroidism during the first 2 years of life. This time course corresponds, grossly, to the weaning period in Ubangi (2). After 3–4 years, the prevalence of goiter and of biochemical hypothyroidism (elevated TSH and/or decreased T_4) in children of the treated group was as elevated as that in children of the control group.

The mean levels of urinary iodine concentration in neonates and in infants of the treated

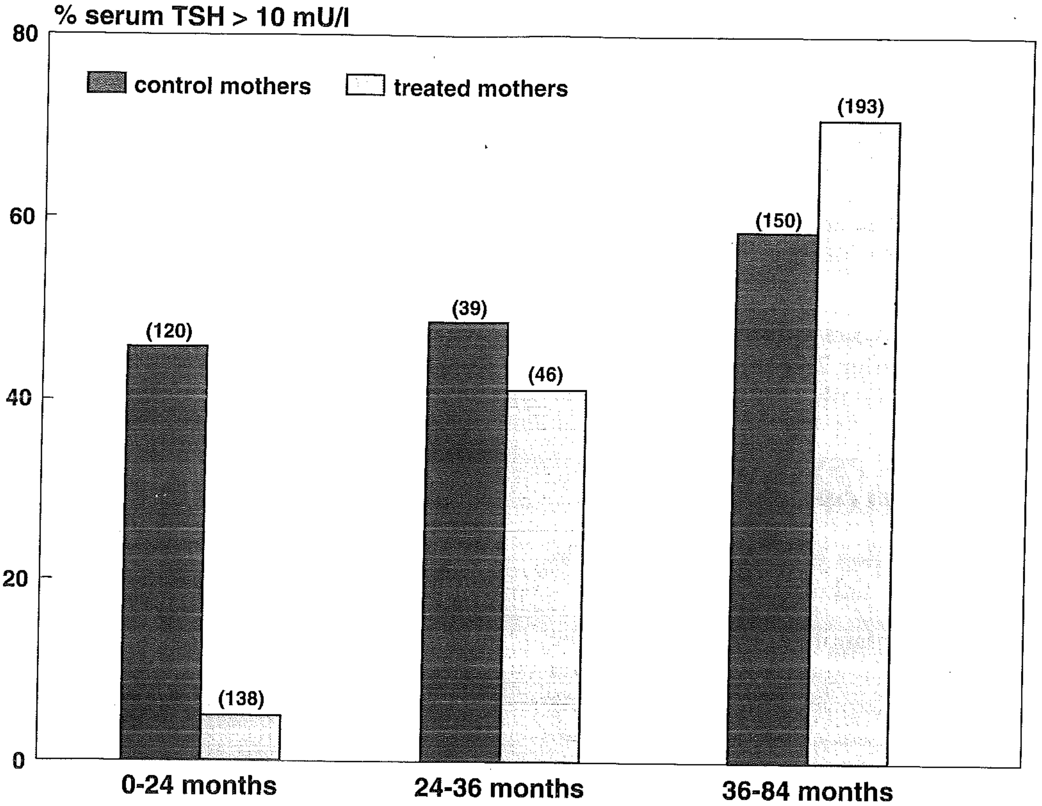

Figure 3. Evolution of the prevalence of elevated serum TSH values in the children of treated mothers and in the children of control mothers.

group are in an elevated normal range (15 μg/dL in the first semester), but well below toxicity (>100 μg/L). This could explain that cases of neonatal hypothyroidism associated with iodine overload were not observed in this study. On the other hand, the relatively short period of protection of iodine deficiency in the child (2 years) as compared to the longer action of iodized oil in the adult (≥4 years) could be easily explained by the fact that, in the child, iodine is probably transferred via the maternal milk in an inorganic form, and iodized oil administered to the adult forms a slowly resorbable deposit.

In the control group, endemic neonatal hypothyroidism is frequently transient, as many children have recovered normal thyroid function in the absence of iodine supplementation; on the other hand, normal thyroid function at birth does not predict a normal thyroid function during childhood. Neonatal, infantile, and juvenile hypothyroidism affects the child at some stage of development with variable severity, with variable duration, and with variable time of onset for the majority of children of Ubangi. The administration of iodized oil to the mother protects the neonate and infant until 2 years; after that time, the risk of juvenile hypothyroidism is as great in the treated group as in the control group.

Only part of biologically hypothyroid children are classified as cretins. They represent children affected by long-standing hypothyroidism resulting in a characteristic phenotype (intellectual impairment, stunted growth, retardation of bone maturation). In the control group, the age of installation of long-standing hypothyroidism in cretins is also variable, and, if already present during the first 2 years, it involves irreversible psychomotor development. Iodized oil during pregnancy certainly prevents the installation of a long-standing hypothyroidism during the first 2 years, that is, during a critical period of brain development dependent on the thyroid hormonal status. It does not prevent long-standing hypothyroidism after weaning, with its characteristic detrimental effects on statural development; at least this treatment protects psychomotor development (vide infra).

In conclusion, the administration of iodized oil (1 cc Lipiodol IM) to the mother during the last two trimesters of pregnancy is safe, and efficiently protects the thyroid function of the off-

Table 3. Distribution of Psychomotor Development Scores

Psychomotor Development Score	≤0.60	0.60–0.80	≥0.80
Control group	9.7% (17/175)	17.1% (30/175)	73.1% (128/175)
Treated group	0.5% (1/218)	20.2% (44/218)	79.4% (173/218)
		χ^2 (2 df) = 19.1 ($p < 0.001$)	

Scores in children of mothers treated with iodized oil during pregnancy (treated group) or with placebo (control group). χ^2 (2 df): chi-square test with two degrees of freedom.

spring during the first 2 years. Iodine supplementation should be repeated after 2 years in the children to prevent juvenile hypothyroidism.

EFFECTS ON PSYCHOMOTOR DEVELOPMENT

The period of protection of iodine deficiency in this protocol has two main characteristics.

1. Iodine supplementation does not cover the first trimester of pregnancy, a critical period of prenatal neurological development during which iodine deficiency may involve the characteristic clinical defects of endemic neurological cretinism (4–6);
2. Iodine supplementation covers the first 2 years of life, a critical period of neurological development during which hypothyroidism results in irreversible intellectual defects.

The question arises, then, whether iodine supplementation during the last two trimesters of pregnancy is efficient and sufficient to prevent iodine-deficiency associated intellectual impairment in the children. According to the protocol, only the defects associated with postnatal hypothyroidism are prevented, and not those associated with iodine deficiency during the first or second trimester of fetal life. Which are the relevant differences between the treated group and the control group in psychomotor development?

Methods

Psychomotor development was assessed during two of the 11 surveys of the study of Karawa. During the first survey (November 1975), the tests of Gesell modified by Brunet–Lezine were slightly adapted to the cultural environment of Ubangi by a psychologist (7). During the second survey (March 1980), these adapted tests were used by a general practitioner with translation of the questions in the local language by a nurse.

In a few words, these tests contain items of language development, motor development, adaptability, and sociability for different age groups from 1 month to 7 years. The tests were performed in 20 min/child. Analysis of the data of the first survey of psychomotor development has already been published (7). The global score of psychomotor development was determined by dividing the psychomotor development age with the chronological age (a mean score of 1.0 is expected in a normal population). This section presents the analysis of the second survey of psychomotor development (March 1980).

Results

The effect of iodized oil supplementation to the pregnant mother on the psychomotor development of the child is shown in Figure 4. In the treated group, the mean psychomotor development score is stable with age, and slightly lower than the expected mean value for normal (1.0). In the control group, the trend of the mean psychomotor development score to decrease with age is not significant. However, when the four age classes are regrouped, the mean psychomotor development score is lower in the control group (0.82 ± 0.14, $n = 175$) than in the treated group (0.91 ± 0.13, $n = 218$) ($p < 0.05$).

Table 3 shows the proportions of subjects with a psychomotor development score of ≤ 0.60, > 0.60, < 0.80; and ≥ 0.80. With one exception (0.5%, 1/218), the low psychomotor development scores (≤0.60) are observed only in the control group (9%, 17/175) (control vs. treated: $p < 0.001$). Analysis of the individual cases shows that the unique case of the low psychomotor development score in the treated group is clinically and biologically (TSH < 10 mU/L; $T_4 > 6$ µg/dL) euthyroid. The analysis of the 17 cases of low psychomotor development scores in the control group shows that eight cases were hypothyroid clinically and biologically (one of them being also deaf-mute); nine cases were defined clinically as euthyroid, and showed a normal bi-

Table 4. Comparison of Psychomotor Development Scores

Control Group	Psychomotor development score of the child		
	<0.60	0.60–0.90	>0.90
Prenatal maternal serum TSH			
<10 mU/L	6% (4/62)	29% (18/62)	65% (40/62)
≥10 mU/L	23% (7/30)	27% (8/30)	50% (15/30)
			χ^2 (2 df) = 5.57 (p = 0.06)
**Treated Group**	**Psychomotor development score of the child**		
	<0.60	0.60–0.90	>0.90
Prenatal maternal serum TSH			
<10 mU/L	1% (1/103)	38% (39/103)	61% (63/103)
≥10 mU/L	0% (0/13)	38% (5/13)	62% (7/13)
			χ^2 (2 df) = 1.23 (ns)

Scores in children of mothers treated with iodized oil during pregnancy (treated group) or with placebo (control group), according to the prenatal serum TSH value in the mother before administration of treatment. χ^2 (2 df): chi-square test with two degrees of freedom. NS, not significant.

ology in two cases, altered biology in three cases, and no biological data in four cases. This table shows also that moderate intellectual deficit (psychomotor development between 0.60 and 0.80) was as frequent in the control group (17.1%, 30/175) as in the treated group (20.2%, 44/218) (not a statistically significant difference).

Table 4 compares the distribution of psychomotor development scores in the children divided into the control group and in the treated group according to the prenatal maternal serum TSH. In the control group, hypothyroid mothers were at greater risk to have a child with a very low psychomotor development score (<0.60). Such a detrimental effect of maternal hypothyroidism on psychomotor development of the child was prevented by administration of iodized oil.

Figure 5 shows the individual scores of psychomotor development in the cretins of the longitudinal Karawa study who were given the

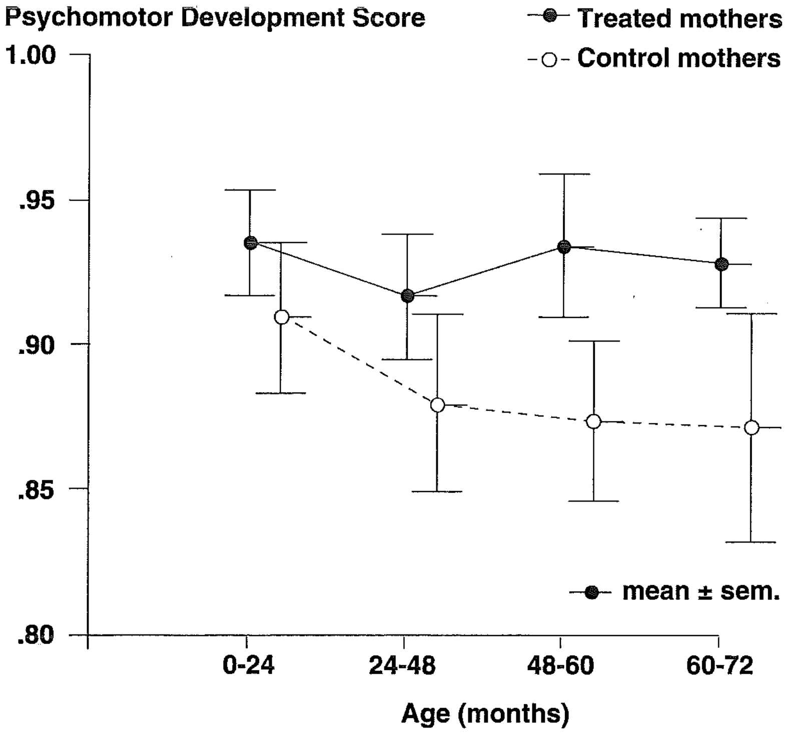

Figure 4. Evolution of the mean psychomotor development score (±1 SEM) in the children of the treated mothers and in the children of the control mothers.

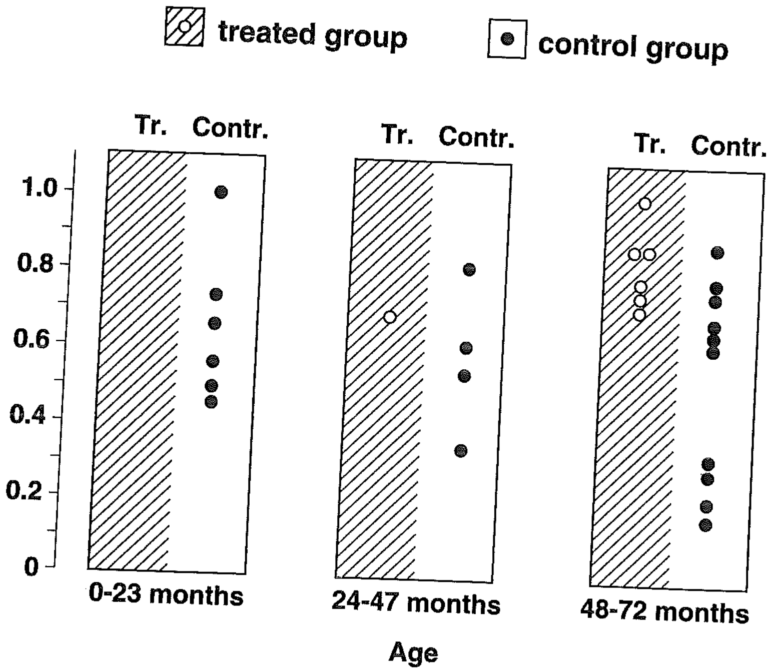

Figure 5. Individual psychomotor development score values in cretins of mothers treated during pregnancy with iodized oil and in control mothers.

iodized oil supplementation during pregnancy. In the control group, 20 scores were determined, and 14 of them were lower than 0.7. In the treated group, fewer cretins were diagnosed (vide supra), and in the 7 cases for whom a psychomotor development score was available, it was >0.7 in every case (chi-square test with Yates correction: $p = 0.006$ vs. control group).

Discussion

Psychomotor development depends on a normal thyroid status of the mother during the first trimester (neuronal proliferation influenced by the maternal thyroid status) and on a normal thyroid status of the fetus at the end of pregnancy and during the first 2–3 years of postnatal life [proliferation and differentiation of glial cells (5)]. Clinical sequelae of hypothyroidism during either period are irreversible. The psychomotor development tests (Gesell tests) would detect the defects associated with first trimester fetal life (coordination, psychomotricity) as well as those associated with early postnatal life (general intelligence, conceptualization). Because iodine supplementation in the present protocol occurred after the first trimester of pregnancy, a comparison of psychomotor development in treated and control groups measures essentially the effect of iodine deficiency in early postnatal life.

A markedly delayed psychomotor development (score < 0.60) was observed mainly in the control group (17 cases). Eight of these cases were diagnosed as hypothyroid cretins (one of them being a mixed form of hypothyroid and neurological cretin, as he was deaf-mute). These eight cases were probably hypothyroid since birth, and represent the most severe form of hypothyroid cretinism. Three cases had an altered biology without a diagnosis of cretinism and two cases were euthyroid. Various possibilities arise: some of them may be mentally retarded for causes independent of iodine deficiency; some of them may be transiently hypothyroid around birth with irreversible intellectual impairment, but they have recovered a normal thyroid function later (transient endemic neonatal and infantile hypothyroidism); some of them may be misclassified as noncretins at a young age, the full spectrum of cretin phenotype becoming clinically evident at a later age. Inversely, children classified as cretins were not all severely mentally retarded. Peculiarly, in the treated group, that is, when the child has probably not suffered from hypothyroidism during the first 2 years, the psychomotor development score was relatively good (>0.7); the diagnosis of cretinism in these cases was based on evidence of hypothyroidism associated with stunted growth and physical sequelae of long- standing hypothyroidism (typical morphotype).

In the control group, when the mother is hypothyroid during pregnancy, the child is at greater

risk to be severely mentally retarded. This association was not observed in the treated group: this lack of association of psychomotor development in the child with thyroid status of the mother during pregnancy in the treated group is a strong argument that neurological defects in the cretin of Zaire (mainly hypothyroid cretinism) differ from those in the cretin of New Guinea (neurological cretinism), where iodized oil is effective in the prevention of neurological defects only if it is administered very early in pregnancy (4). Concerning the myxedematous cretin of Zaire, there is no need to postulate any other mechanism than thyroid failure to explain the mental retardation (8).

Nevertheless, the elevated frequency of moderate psychomotor development retardation in Zaire (between 0.60 and 0.80) suggests that iodine deficiency during the first trimester of pregnancy has involved moderate irreversible neurological defects in some subjects not corrected by the administration of iodized oil during the second and third trimesters (the frequencies of these defects are similar in the control and in the treated group). To confirm this hypothesis, other types of protocols (administration of iodized oil at various periods of pregnancy, including the first trimester) are required. More subtle neurological examination by more experienced pediatric neurologists should help to discriminate the neurological defects associated with iodine deficiency during the first trimester of pregnancy and the early postnatal life and could also contribute to determining the respective effects of iodine deficiency on neurological development in pregnancy and in early postnatal life (9).

CASE-CONTROL STUDY: COMPARISON OF CRETIN AND NON-CRETIN HYPOTHYROID CHILDREN

Endemic neonatal, infantile, and juvenile hypothyroidism is frequent in Ubangi, affecting most of the children at some time of development. Only a minority of these children are classified as hypothyroid cretins.

Which criteria differentiate the young hypothyroid cretins of Ubangi and the many other noncretin hypothyroid children?

To answer this question, a case-control analysis was conducted in which the following parameters were compared in hypothyroid cretins, noncretin hypothyroid children, and normal children:

- the severity of biochemical hypothyroidism;
- the severity of physical growth retardation (height, bone age);
- the severity of psychomotor development retardation;
- the severity of loss of thyroid functional capacity.

Another problem arises: in the longitudinal study of Karawa, the cretins are relatively young (less than 7 years). Are these young cretins evolving to the same clinical picture at adult age as the adult hypothyroid cretins described previously by other investigators (10,11)? To answer this question, the natural evolution of physical growth (height, bone age) in young cretins (0–18 years) diagnosed in the eighties is compared to the items (height, bone age) obtained in adult cretins by these previous investigators (10,11).

Methods

From the control group of children included in the longitudinal Karawa study on iodized oil supplementation in pregnancy, it was possible to extract data on baseline thyroid function (serum TSH, T_4, T_3), physical development (height), and psychomotor development in cretins, hypothyroid children, and euthyroid children less than 7 years (vide infra for the definitions of cretins, hypothyroid children, and euthyroid children).

To this data base, other data collected by three examiners from 1978 to 1985 during various protocols were added. The subjects were classified according to the same criteria in cretins, hypothyroid children, and euthyroid children. From these data bases, it was possible to extract data on:

- baseline thyroid function (serum TSH, T_4, T_3);
- thyroid functional capacity (variation of serum thyroid hormone after iodide supplementation (20 μg KI/kg per os);
- physical development (height);
- bone maturation: radiological age (X-ray of the wrist and the knee) determined with the tables of Pyle and Hoerr (12) and with the tables of Greulich and Pyle (13);
- psychomotor development determined for the subjects less than 7 years by Gesell tests modified by Brunet–Lezine and adapted to rural Africa by Roger (7).

Cretinism (hypothyroid cretinism or mixed cretinism, hypothyroid cretinism with neurological

Table 5. Distribution of Thyroid Function Hormonal Values

	Biology		
	Euthyroid Hormonal Values	Moderately Hypothyroid Hormonal Values	Severly Hypothyroid Hormonal Values
	Serum TSH ≤ 10 mU/L, $T_4 \geq 6$ μg/dL	Serum TSH > 10 mU/L, $T_4 > 5$ μg/dL or Serum TSH < 40 mU/L, $T_4 < 6$ μg/dL	Serum TSH ≥ 40 mU/L, $T_4 \leq 5$ μg/dL
Cretins (n = 235)	16 (6.8%)	37 (15.7%)	182 (77.4%)
Noncretin subjects (n = 591)	260 (44.0%)	156 (26.4%)	175 (29.6%)

Values in three classes (euthyroid; moderately hypothyroid; severely hypothyroid) in subjects clinically classified as cretins or as noncretin subjects.

defects was defined with clinical and biochemical criteria (serum $T_4 \leq 5$ μg/dL and serum TSH > 40 mU/L). Hypothyroid children were defined with clinical criteria (classified as noncretin) and with biochemical criteria ($T_4 \leq 5$ μg/dL and TSH ≥ 40 mU/L). Euthyroid children were defined on clinical criteria (noncretin) and on biochemical criteria ($T_4 \geq 6$ μg/dL and TSH ≤ 10 mU/L). Cretins and noncretin children with intermediate biological values ($T_4 < 6$ μg/dL and TSH < 40 mU/L or $T_4 > 5$ μg/dL and TSH > 10 mU/L) were excluded from the analysis.

Results

Table 5 shows the distribution of thyroid function hormonal values (normal, moderately hypothyroid, severely hypothyroid) in cretins and noncretin subjects. Three quarters (77.4%, 129/235) of the cretins were severely hypothyroid versus 29.6% (175/591) of the noncretin subjects ($p < 0.001$). Some cretins had no hypothyroidism at the time of examination (6.8%, 16/235), versus 44% (260/591) biologically euthyroid values in the noncretin subjects ($p < 0.001$).

Table 6 compares the baseline thyroid function hormonal values, the thyroid function hormonal values after administration of iodine, the physical growth, and the psychomotor development in cretins, hypothyroid children, and euthyroid children. By definition, cretins and hypothyroid children were severely hypothyroid (serum TSH ≥ 40 mU/L and serum $T_4 \leq 5$ μg/dL). Mean serum TSH was slightly more elevated in cretins than in hypothyroid subjects; the mean serum T_3 level was lower ($p < 0.001$) and the proportion of low serum T_3 concentrations was greater ($p < 0.001$) in cretins than in hypothyroid subjects. The response to iodine supplementation was incomplete in cretins, 74% of them (17/24) remaining with an elevated TSH (>20 mU/L) 1 week after iodine administration. The proportion of incomplete response to iodine supplementation was markedly lower in noncretin hypothyroid subjects (14%, 2/14) ($p < 0.001$). The physical development was more delayed in cretins than in hypothyroid subjects: 58% (86/149) of the cretins versus 20% (25/123) of the noncretin hypothyroid subjects were under 80% of the median reference height for age ($p < 0.001$) and 76% (59/78) of the cretins versus 50% (23/46) of the noncretin hypothyroid subjects had a bone age less than half their chronological age (bone maturation score < 50%) ($p = 0.006$). It was also notable that the physical development of hypothyroid subjects was delayed when compared with the euthyroid subjects. Cretins had a delayed psychomotor development as compared to hypothyroid subjects; the psychomotor development of these last subjects was similar to that of euthyroid subjects.

From Table 6 it is possible to calculate the likelihood ratio (LR) of being a cretin versus a noncretin hypothyroid subject; this LR is a good index of the discriminant value of a parameter. For example, the serum T_3 value is <120 ng/dL in 61% of the cretins and in 17% of the noncretin hypothyroid subjects; the LR for serum T_3 (cretin vs. hypothyroid subject) is 3.6 (61%/17%) (a subject with a serum T_3 < 120 ng/dL had 3.6 times more risk of being a cretin than a noncretin hypothyroid subject). If one calculates the LR's for the other variables in cretins versus hypothyroid subjects, in decreasing order of importance:

LR serum TSH > 20 mU/L after KI = 5.3 (74%/14%)
LR psychomotor development score < 0.80 = 4.2 (72%/17%)
LR basal serum T_3 < 120 ng/dL = 3.6 (61%/17%)
LR height < 80% median = 2.9 (58%/20%)
LR bone maturation < 50% = 1.5 (76%/50%).

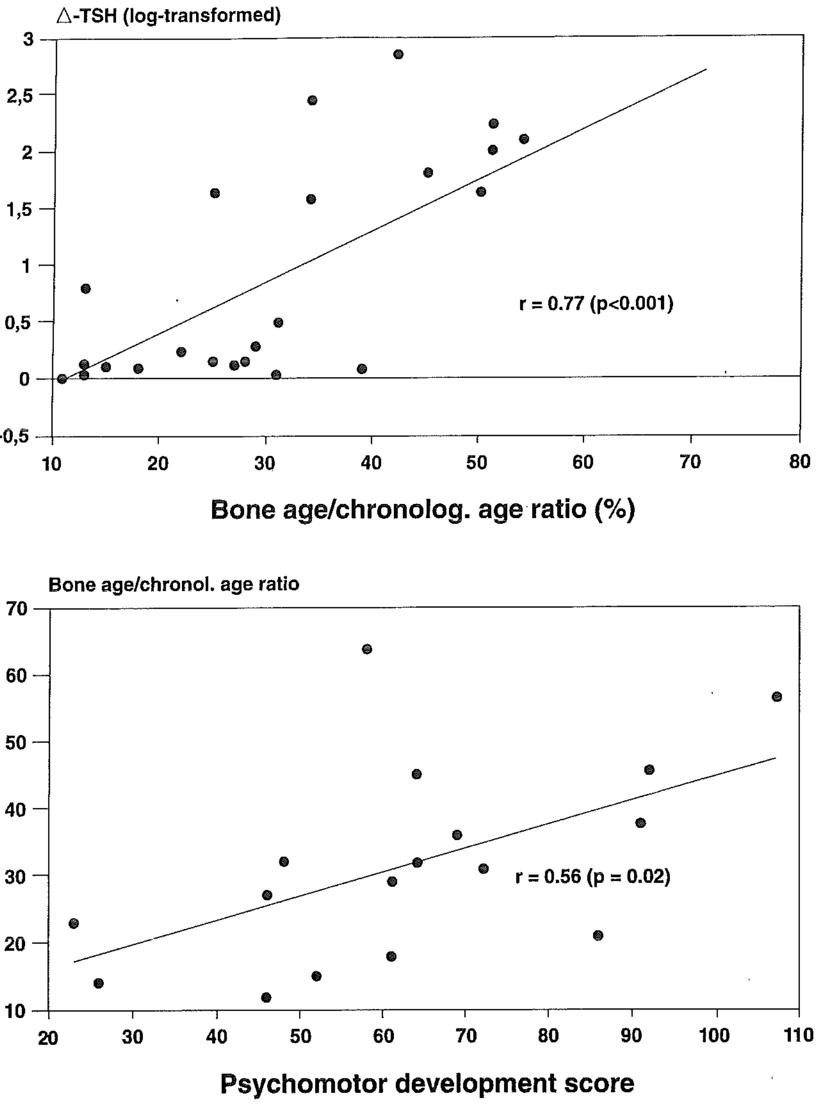

Figure 6. Top: relationship of the variation of serum TSH in cretins after iodine supplementation (7 days after 20 μg KI per os once) with bone age maturation. Bottom: relationship of bone age maturation with psychomotor development in cretins.

Figure 6 (top) presents the relationship, in cretins, of the thyroid functional capacity (proportional to the variation of serum TSH after KI) with the bone maturation ratio. The severity of bone maturation retardation was closely associated to the severity of thyroid functional capacity involution, as expressed by the low variation of serum TSH. A significant positive relationship (Figure 6, bottom) was also observed between bone age and psychomotor development.

To determine whether the cretins examined in this study were similar to those described previously by other investigators in Idjwi Island, Kivu Lake (11) or in Uélé, Northern Zaire (10), the distribution of heights with age in cretins of this study was compared to the distribution of heights in adult cretins previously reported. From Figure 7, it appears that the extrapolated distribution of adult height in cretins of this study is superimposed on the distribution of adult height in cretins of previous studies.

Discussion

The present data compare thyroid function, physical development, and psychomotor development in cretins, in biochemically hypothyroid subjects not classified as cretins, and in clinically and biochemically euthyroid subjects.

As expected, cretins are more hypothyroid (more elevated mean serum TSH concentration, lower mean serum T_3 concentration), have less thyroid functional capacity (lower decrease of mean serum TSH after KI administration), have more physical growth retardation (height, bone age), and are more mentally retarded than noncretin hypothyroid subjects. Within the large proportion of subjects affected by hypothyroidism,

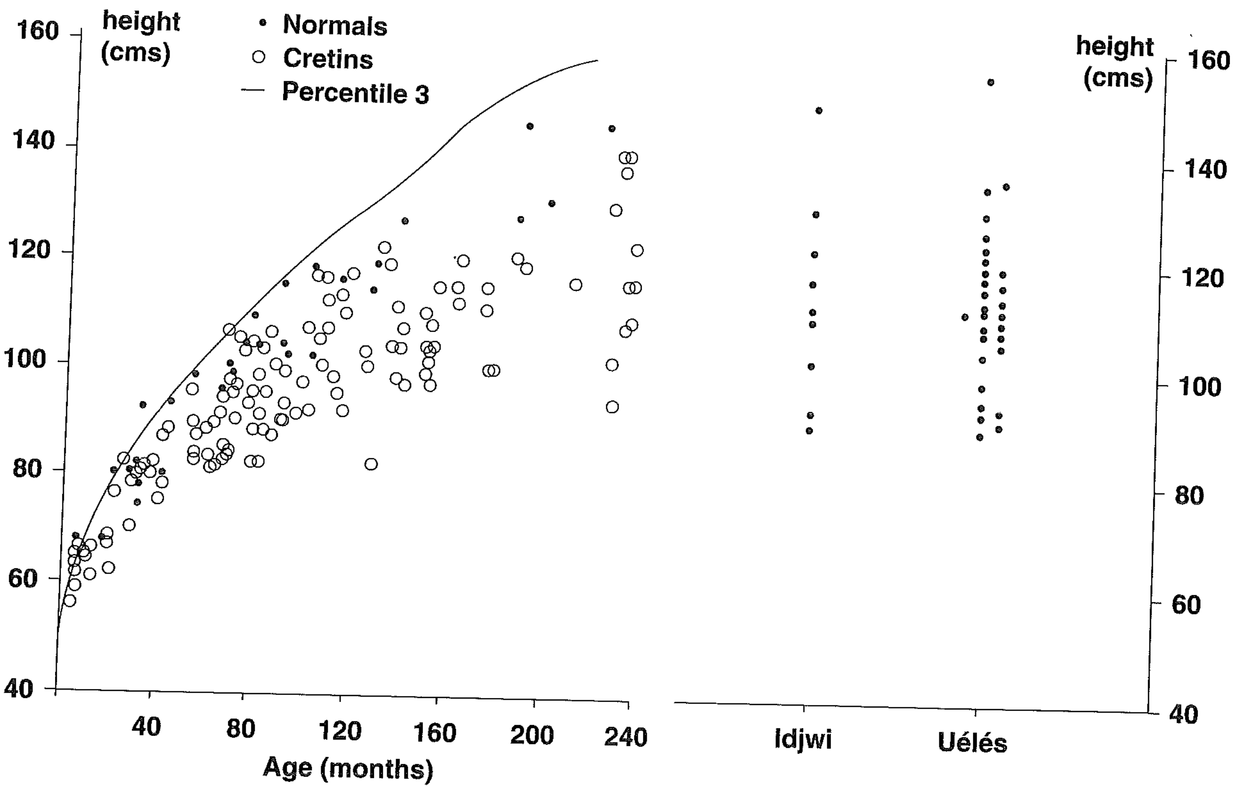

Figure 7. Distribution of the height with age in cretins and in normal subjects of Ubangi. The continuous line represents the Percentile 3 of the height in reference tables. On the right, the distribution of the heights of adult cretins of Idjwi Island, Kivu Lake (11) and of Uélé, Northern Zaire (10) is plotted.

some children show evident signs of long-standing hypothyroidism after birth with its clinical sequelae (mental deficiency, stunted growth). The main difference between cretins and noncretin hypothyroid subjects is then associated with the variable time of onset and the variable duration of hypothyroidism in early childhood (long-standing vs. transient) (Figure 8). As previously shown (14) and as confirmed in other endemic areas (15) and in a larger series in Zaire (16), long-standing hypothyroidism in iodine-deficient children is associated with a progressive involution of thyroid function. Endemic neonatal, infantile, and juvenile hypothyroidism involves a loss of responsiveness to iodine supplementation, and this initially potentially transient state becomes irreversible. The involution of thyroid function explains the mean lower serum T_3 concentration in cretins versus hypothyroid subjects. The clinical features of juvenile hypothyroidism are not obvious during the early period of hypothyroidism, and it is not surprising that the growth and psychomotor development in most noncretin hypothyroid subjects do not differ markedly from those in euthyroid subjects. In contrast, most cretins have a height, bone maturation, and psychomotor development clearly lower than those in hypothyroid children or in euthyroid children.

However, the overlap of individual values between these two groups is great. When the baseline thyroid function hormonal values are analyzed, there is no clear cut difference between cretins and hypothyroid subjects. It is not possible to define a threshold of serum TSH, serum T_4, and serum T_3 that discriminates between these two groups. Although mean serum T_3 is markedly lower in cretins than in hypothyroid subjects, only 61% of the cretins are under the normal limit (<120 ng/dL); 17% of the hypothyroid subjects also have a serum T_3 under this limit. In other words, baseline serum thyroid function hormonal values are largely overlapping in cretins and in noncretin hypothyroid subjects. The definition of endemic cretinism has an epidemiological usefulness for assessing the severity of an endemia. However, this clinical definition has poor sensitivity and poor precision because it does not take into account the physical and intellectual defects in a large number of subjects who are transiently hypothyroid. This clinical definition also lacks specificity be-

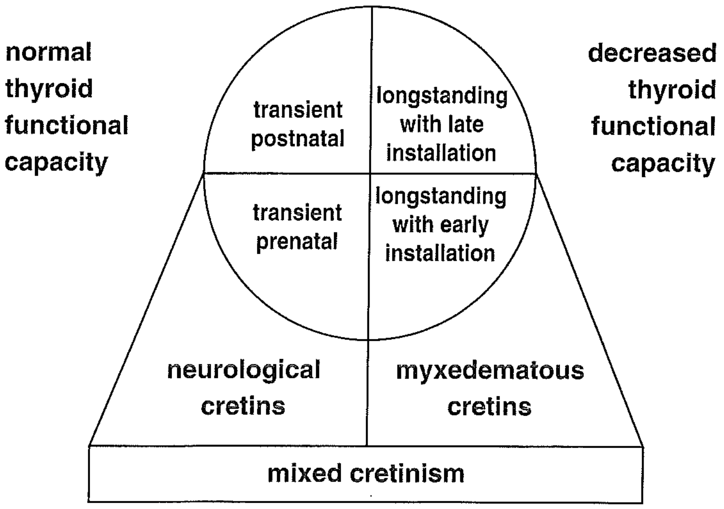

Figure 8. Proposed model of pathogenesis of endemic cretinism. Endemic hypothyroidism may be transient or long- standing. In the case of transient feto–maternal hypothyroidism occurring during the first trimester of pregnancy, irreversible damage to the neurological development involves neurological cretinism. In the case of long-standing hypothyroidism, the functional capacity of the thyroid gland decreases, and hypothyroidism becomes irreversible, even after iodine supplementation (endemic myxedematous cretinism). If hypothyroidism affects the feto–material unit during early fetal life and persists during childhood, neurological defects are superimposed on irreversible hypothyroid cretinism (endemic mixed cretinism).

cause many subjects classified as cretins are not distinguishable from the rest of the population when objective measurements are performed.

How can we assume that the young cretins in this study have the same defects as those described previously by other investigators (10,11)? Are there arguments to suggest that the criteria to define cretinism in this study were less restrictive than in previous studies? In the previous studies, adult cretins were selected on the basis of evidence of the complete clinical features of endemic myxedematous cretinism. This kind of selection recruits the cases with the most severe forms of cretinism. In the present study, children were examined within a cohort of normal subjects, and each child was classified as cretin or noncretin; this kind of selection gives a more accurate picture of the frequency of cretinism in a population, but the difficulty arises in classifying accurately the subjects on clinical criteria. Despite the differences in the mode of selection and in the age of cretins, when one extrapolates the expected distribution of height in adulthood from the distribution of heights in young cretins of this study, it is superimposed on the distribution of adult heights recorded in previous studies (30 and 21 years ago). This argument is also valid for bone maturation and psychomotor development (17). The cretins of this study were similarly affected by long-standing hypothyroidism as those previously described.

In conclusion, the usual clinical definition of cretinism in the field has no clear-cut criteria, and this definition includes, in practice, a large variety of subjects affected by various degrees of physical and intellectual defects. The full spectrum of persistent hypothyroidism, mental deficiency, stunted growth of the typical morphotype is found in two thirds of the subjects classified as cretins; the other third is represented, at least in part, by subjects who have either mental retardation with a normal growth, or a physical development retardation with a normal intellect. The overlap is important between cretins and noncretin hypothyroid subjects, whatever the criteria used.

SUMMARY

1. Hypothyroidism is very frequent in early life in severe endemic goiter areas. The variable period of installation, duration, and severity of hypothyroidism explains the variable physical and psychomotor sequelae. Long-standing endemic neonatal and juvenile hypothyroidism is associated with a decrease of thyroid functional capacity, evolving through an irreversible state of hypothyroidism.
2. In a placebo-controlled, longitudinal, randomized study, iodized oil administered during the two last trimesters of pregnancy prevents hypothyroidism during a critical phase of 2 years. This period corresponds, grossly, to the weaning period. After 3 years, the sever-

ity of hypothyroidism in children of treated mothers is similar to that in children of control mothers.

3. Iodized oil administered during the last two trimesters of pregnancy drastically reduces the incidence of severe psychomotor development retardation (score < 0.60) (0.5% in treated group vs. 9% in control group). It cannot be excluded that the poor psychomotor performances (psychomotor development score between 0.60 and 0.80) in a significant proportion of children of the treated group and of the control group were the result of feto-maternal hypothyroidism during the first trimester.
4. Only a minority of children with biochemical hypothyroidism (TSH $\geq$ 40 mU/L and $T_4 \leq 5$ μg/dL) were classified as cretins on the basis of clinical criteria. When compared to noncretin hypothyroid children and to euthyroid children, cretins had a lower mean serum T_3 concentration, a decreased responsiveness to iodine supplementation, a delayed physical development (height, bone maturation), and a lower psychomotor development. When compared to euthyroid children, noncretin hypothyroid children had a similar mean serum T_3 concentration, a delayed physical development; their thyroid responded normally to iodine supplementation. Nevertheless, no clear-cut criteria: of thyroid function hormonal parameters; of responsiveness to iodine supplementation; of physical development; or of psychomotor development, discriminates cretins, noncretin hypothyroid subjects, and euthyroid subjects. It is likely that most of the children suffer at some time from hypothyroidism during critical phases of physical and psychomotor development (endemic transient neonatal and juvenile hypothyroidism). If hypothyroidism persists for a long period, it becomes irreversible as a result of an involution of thyroid function (endemic long-standing neonatal and juvenile hypothyroidism leading to endemic myxedematous (hypothyroid) cretinism.
5. Iodized oil during the last two trimesters of pregnancy prevents the most severe cases of myxedematous cretinism beginning around birth; it does not prevent hypothyroidism beginning after weaning. It is not excluded that long-standing juvenile hypothyroidism of late installation (after weaning) can still be associated with a progressive loss of thyroid functional capacity. To prevent juvenile hypothyroidism, iodine supplementation in children of mothers treated during pregnancy with iodized oil should be repeated after 2 years.

REFERENCES

1. Thilly, C.; Delange, F.; Lagasse, R.; et al. Fetal hypothyroidism and maternal thyroid status in severe endemic goiter. J. Clin. Endocrinol. Metab. 47:354–360; 1978.
2. Vanderpas, J.; Bourdoux, P.; Lagasse, R.; et al. Endemic infantile hypothyroidism in a severe endemic goiter area of Central Africa. Clin. Endocrinol. 20:327–340; 1984.
3. Bourdoux, P.; Seghers, Ph.; Mafuta, M.; et al. Biochemical and statistical methods., In Delange, F.; Iteke, F.B.; Ermans, A.M., Eds. Nutritional factors involved in the giotrogenic action of cassava, International Development Research Centre (IDRC), Ottawa, Canada; 51–58; 1982.
4. Pharoah, P.O.D.; Buttfield, I.H.; Hetzel, B.S. Neurological damage to the fetus resulting from severe iodine deficiency during pregnancy. Lancet 1:308–310; 1971.
5. Morreale de Escobar, G. Thyroid hormones and the developing brain. In Medeiros–Neto, G.; Gaitan, E., Eds. Frontiers in thyroidology, Vol. 1, Plenum Medical Book Company, New York: 5–19; 1986.
6. Hetzel, B.S. An overview of the prevention and control of iodine deficiency disorders. In Hetzel, B.S.; Dunn, J.T.; Stanbury, J.B., Eds. The prevention and control of iodine deficiency disorders, Elsevier Science Publishers Biomedical Division, Amsterdam; 7–31; 1987.
7. Roger, G. Evaluation du développement psychomoteur de jeunes enfants vivant en Ubangi (Nord-Zaïre) zone de sévére endémie goitreuse. Rev. Internat. de l'Enfant 44:45–50; 1980.
8. Corvilain, B.; Contempré, B.; Longombe, A.O., et al. Selenium and the thyroid: How the relationship was established. Am. J. Clin. Nutr. 57 (Suppl.): 244S–248S; 1993.
9. DeLong, R. Neurological involvement in iodine deficiency disorders. In Hetzel, B.S.; Dunn, J.T.; Stanbury, J.B., Eds. The prevention and control of iodine deficiency disorders, Elsevier, 49–63; 1987.
10. Dumont, J.E.; Ermans, A.M.; Bastenie, P.A. Thyroid function in a goiter endemic. IV. Hypothyroidism and endemic cretinism. J. Clin. Endocrinol. Metab. 23:325–335; 1963.
11. Delange, F.; Ermans, A.M.; Vis, H.L.; Stanbury, J.B. Endemic cretinism in Idjwi island (Kivu Lake, Republic of the Congo). J. Clin. Endocrinol. 34: 1059–1066; 1972.
12. Pyle, S.I.; Hoerr, N.L. Radiographic atlas of skeletal development of the knee. Thomas, Springfield, IL; 1955.
13. Greulich, W.W.; Pyle, S.I. Radiographic atlas of skeletal development of hand and wrist. Stanford University Press, Stanford; 1959.

14. Vanderpas, J.; Rivera, M.T.; Bourdoux, P.; et al. Reversibility of severe hypothyroidism with supplementary iodine in patients with endemic cretinism. N. Engl. J. Med. 315:791–795; 1986.
15. Boyages, S.C.; Halpern, J.P.; Maberly, G.F.; et al. Supplementary iodine fails to reverse hypothyroidism in adolescents and adults with endemic cretinism. J. Clin. Endocrinol. Metab. 70:1341–1346; 1990.
16. Vanderpas, J.; Contempré, B.; Duale, N.J.; et al. Selenium deficiency mitigates hypothyroxinemia in iodine- deficient subjects. Am. J. Clin. Nutr. 57 (Suppl.):271S–275S; 1993.
17. Vanderpas, J., L'hypothyroïoïdie juvenile endémíque en Ubangi. Zaïre. Thesis, Université Libre de Bruxelles, 1–139; 1991.

Acknowledgements:Data included in this report were obtained in part during surveys in the field by many colleagues. Special thanks to Dr. François Delange, pediatrician (survey, 1974), to Dr. Raphaël Lagasse and Mrs. Geneviève Roger (survey, 1975), to Dr. Jan Smitz, clinical pathologist (survey, 1983), and to Dr. Henri Deckx (survey, 1984). The collaboration of the medical staff of the hospital of Karawa, Zaire, is greatly appreciated (Drs. Roger Thorpe, Helen Berquist, Leo Lanoie, Mrs. Grace Nelson, RN). The help of the Institut de la Recherche Scientifique, Zaïre (Dr. Y. Yunga, Mr. Bokango, RN) was essential. The biological measurements were performed in the laboratory of the Hôpital Universitaire Saint-Pierre at in Brussels (director: Dr. André-Marie Ermans) under the supervision of Mr. Pierre Bourdoux, Ph.D. The assistance in the field and the technical skill for for biological measurements of Maria Teresa Rivera are recognized.

Financial support by the Program «Science et Technique au Service du Développement» from the Commission of the European Community, contract ECC-STD DGXII TS3*-CT92-01371 and by Grant Nr. 3.4530.93 from the «Fonds de la Recherche Scientifique Médicale, FRSM», Belgium.

Table III-2. Comparison of thyroid function hormonal concentrations at baseline and after KI administration, of physical development and of psychomotor development in cretins, non-cretin hypothyroid subjects, and euthyroid subjects of Ubangi, Northern Zaire.

	CRETINS		HYPOTHYROID SUBJECTS		EUTHYROID SUBJECTS	
**baseline thyroid function hormonal concentrations (age: 0-38 yrs)**						
mean serum TSH (mU/l) (geom. mean, -/+ 1 SD)	218 (102-469)	(182)^{f,c}	156 (70-345)	(156)^{c}	2.1 (0.7-5.8)	(260)
mean serum T4 (µg/dl) (± 1 SD)	1.2 ± 1.1	(182)^{c}	1.6 ± 1.2	(156)^{c}	11.8 ± 3.6	(260)
mean serum T3 (ng/dl) (± 1 SD)	115 ± 71	(178)^{f,c}	198 ± 75	(152)^{d}	202 ± 64	(248)
% serum T3 < 120 ng/dl (%,n°)	61%	(105)^{f,c}	17%	(26)	9.7%	(24)
**thyroid function hormonal concentrations at 7 days after oral KI administration (20 µg/kg, once) (age: 2.5-15 yrs)**						
mean serum TSH (mU/l) (geom. mean, -/+ 1 SD)	59 (20-176)	(23)^{f,c}	13 (7.8-22)	(14)^{c}	3.8 (1.1-13)	(9)
% serum TSH > 20 mU/l (%, n°)	74%	(17)^{f,c}	14%	(2)	0%	(0)
mean serum T4 (µg/dl) (± 1 SD)	3.9 ± 3.0	(23)^{c}	4.8 ± 1.9	(15)^{c}	9.9 ± 3.7	(9)
mean serum T3 (ng/dl) (± 1 SD)	103 ± 68	(23)^{f,c}	258 ± 70	(6)^{c}	170 ± 23	(4)
**physical development (age: 0-18 yrs)**						
height % of median (mean ± 1 SD)	78 ± 9	(149)^{f,c}	85 ± 6	(123)^{c}	91 ± 5	(223)
< 80% of med. ref. height for age (%, n°)	58%	(86)^{f,c}	20%	(25)^{c}	2.2%	(5)
bone age/chronol. age, % (mean ± 1 SD)	34 ± 17	(78)^{f,c}	53 ± 16	(46)^{f}	65 ± 14	(34)
bone maturation score < 50% (%, n°)	76%	(59)^{e,c}	50%	(23)^{b}	15%	(5)
**psychomotor development (age: 0-7 yrs)**						
mean psychomotor development score (%)	0.62 ± 0.25	(64)^{f,c}	0.91 ± 0.15	(90)	0.93 ± 0.16	(197)
psychomotor development score < 0.80 (%, n°)	72%	(46)^{f,c}	17%	(15)	20%	(39)

b, c: p<0.01; p<0.001 vs. euthyroid subjects
d, e, f: p<0.05; p<0.01; p<0.001 vs. hypothyroid subjects

DISCUSSION:CHAPTERS 19–21

Stein I wonder if either Dr. Bleichrodt or Dr. Connolly would comment, particularly in view of Dr. Bleichrodt's metaanalysis. Has Dr. Bleichrodt discovered in his analysis anything beside general intelligence quotient that would allow us to identify specific brain loci?

Looking at the subtests of IQ, has that been useful in defining the anatomical locations in the brain? Are there specific subtests that can help in localizing damage?

Bleichrodt It should be possible to make connections between anatomical locations in the brain and intellectual aspects measured by specific subtests.

Connolly I have misgivings about Dr. Bleichrodt's use of metaanalysis because of the enormous variability of the studies included. Different definitions, measures, and designs were used. Arguably, they were not all measuring intelligence, and certainly the reliability and validity of these studies is open to question. A metaanalysis is only as good as the material that goes into it.

Escobar How iodine deficient does a population have to be before abnormalities show up in psychometric testing of school children? As far as I know, most studies carried out so far showing decreased mental and motor performance have been performed in populations with very severe iodine deficiency, where cretins were born, or had been reported to be born until recently. However, it would appear very interesting to know whether abnormalities would also be found in populations with a more moderate iodine deficiency. A study performed by Dr. J.A. Munoz in Teruel, Spain, where the goiter endemia is moderate (grade II) showed decreased hearing acuity in the school children with goiter ($p < 0.01$). I believe some studies in Italy also point to decreased performance in some tests in school children from a grade II endemia. Another point which is not clear is whether performance would be improved in school children with the introduction of iodine prophylaxis, which might be the case if part of the damage were caused by cerebral hypothyroidism during childhood, and not to uterine damage as in the case of neurological cretins.

I believe some of the findings reported by Dr. Mitchell for the CH children who do not comply with treatment when they reach puberty are quite illuminating in this respect. If I have understood correctly, as infants their scores started to become worse when they did not comply with treatment and had lower serum T_4 and higher serum TSH values. Scores again improved when they complied with treatment and their T_4 and TSH test results became normal. This means that scores obtained during psychometric testing are sensitive to the thyroid status of the individual at the moment when they are applied, being negatively affected by hypothyroidism, and that they improve when they become euthyroid. Would this apply also to school children living in iodine deficient conditions, with decreased serum T_4, even if they did not suffer irreversible intrauterine damage?

Bleichrodt This is an interesting question. The separate studies are too small to give a reliable answer. It might be possible to use the metaanalytical method, combining the studies that have data on physiological aspects as well as on psychological aspects.

DeLong Dr. Bleichrodt, you showed us these two very nice normal curves. Do your studies in fact show that the iodine deficient group has a normal distribution or is that a mathematical abstraction?

Bleichrodt Indeed, the distributions I showed were purely theoretical. Yet, the distribution based on empirical data from Spain did not differ significantly from a normal distribution.
