# Supplementary material for: Familial Aggregation of Endemic Congenital Hypothyroidism Syndrome in Congo (DR): Historical Data
Source: Nutrients. 2020 Oct 2;12(10):3021. doi: 10.3390/nu12103021 (PMC7601371; doi:10.3390/nu12103021)
Supplement: Supplementary file 1 [file nutrients-12-03021-s001.zip › Document S1 Ethical Committee Approval P2019 576 N A.docx]

**A :**    [jean.vanderpas@skynet.be](mailto:jean.vanderpas@skynet.be)

**Objet :** Approbation étude clinique P2019/576 / N/A

**Format :** HTML

Cher Dr. Jean VANDERPAS,

Le Comité d’Ethique Erasme-ULB a approuvé le 10/12/2019 votre nouvelle proposition de recherche intitulée : « *Data mining (1979-1980) : familial aggregation of endemic cretinism in Norther Congo.* ».

Référence Erasme : P2019/576

Vous recevrez sous peu le courrier d’approbation de cette étude.

Dans le cadre de vos [responsabilités d’investigateur](http://www.erasme.ulb.ac.be/page.asp?id=14316&langue=FR), nous nous permettons de rappeler :

1. qu’une étude qualifiée de rétrospective concerne les dossiers de patients traités antérieurement à la date de soumission du dossier au comité d’éthique,
2. que dans une étude rétrospective, l’exemption au consentement effectif du patient peut être accordée sous 2 conditions :
   1. Votre engagement à vérifier auprès du Service de la Recherche Biomédicale (contact SRB : Tél : +32 (0)2 555 83 51) que le patient n’a pas exprimé son refus à l’utilisation de son dossier médical à des fins de recherche ;
   2. Votre engagement à prendre toutes les mesures nécessaires à la [protection de la confidentialité des données collectées](http://www.erasme.ulb.ac.be/page.asp?id=14311&langue=FR).
3. que nous souhaitons être informé de la date de début effectif de l’étude (date 1^ère^ consultation des dossiers) et être notifié de la clôture de l’étude avec rapport des résultats obtenus.

Tout courrier/courriel de suivi que vous nous transmettrez reprendra bien les références de votre étude.

Le Comité d’Ethique vous souhaite tout le succès possible dans votre entreprise et je vous prie d’agréer, Cher Dr. Jean VANDERPAS, l’expression de mes meilleurs sentiments.

| 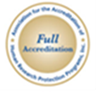 | Hélène François, BSc, PhD  Pour le Comité d'éthique  T +32 (0) 2 555 37 07  T +32 (0) 2 555 37 11 - F +32 (0) 2 555 46 20  M [Comite.ethique@erasme.ulb.ac.be](mailto:Comite.ethique@erasme.ulb.ac.be)  <http://www.hopitalerasme.be/ethique> <http://www.hopitalerasme.be/ethics> |
| --- | --- |
| **Président**: Dr J.-M. Boeynaems  **Vice-présidents**: Dr S. Goldman, Dr H. Louis  **Membres**: Mme B. Abbouz – Mme Alvarez Villamandos – Mr S. Baudry – Mme L. Benammar – Dr J.-C. Bier – Mr A. Counet – Dr. E. Demanet – Mme L. Didens – Dr F. Felgueroso-Bueno – Dr J. Groswasser – Mme. V. Fontaine – Me B. Fonteyn – Mme I. Langer – Dr V. Lesage – Dr G. Loas – Mme Th. Locoge – Dr A. Joosten – Mr M. Mayer – Mr Ph. Peigneux – Mme A. Petiau – Mr Ch. Plomteux – Dr J. Rouby – Dr F. Roufosse – Mme C. Sauvage – Mme S. Scholtes – Dr Ph. Van de Borne – Dr J.-L. Vachiery – Mme C. Verhoeven  **Consultants**: Dr. A. Demols –  Mr G. Niset – Dr F. Vermeulen – Dr B. Byl  **Secrétariat**: Mme F. Martinez, Mme M. Onrubia, Mme C. Piesen  **Contact**: Tél. : 32-2-555.37.07 / 32-2-555.37.11, Fax : 32-2-555.46.20 | |


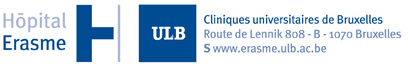


[Lien vers Disclaimer](http://www.erasme.ulb.ac.be/email-disclaimer)
